# New Triterpenoid Saponins from the Herb *Hylomecon japonica*

**DOI:** 10.3390/molecules22101731

**Published:** 2017-10-23

**Authors:** Yan-Fei Qu, Jing-Yu Gao, Jing Wang, Yan-Mei Geng, Yu Zhou, Cheng-Xin Sun, Fei Li, Lei Feng, Meng-Juan Yu, Guang-Shu Wang

**Affiliations:** 1 School of Pharmaceutical Sciences, Jilin University, Changchun 130021, Jilin, China; cream0023@163.com (Y.-F.Q.); gaojingyu1989@126.com (J.-Y.G.); 13578752256@163.com (J.W.); gym130123@163.com (Y.-M.G.); 17623233985@163.com (Y.Z.); 13124303229@163.com (C.-X.S.); 18844194997@163.com (F.L.); xiaoweiaw@163.com (L.F.); 13194312537@163.com (M.-J.Y.); 2 Logistics University of PAP, Tianjin 300162, China; 3 Department of Pharmacy, Central Hospital of Yingkou Economic and Technological Development Zone, Yingkou 115007, Liaoning, China

**Keywords:** *Hylomecon japonica*, *Papaveraceae*, triterpenoid saponin, gypsogenin, quillaic acid

## Abstract

*Background*: *Hylomecon japonica*, a plant of the *Papaveraceae* family which is well-known for the alkaloids they produce, is a perennial plant widely distributed in the northeast, central and east regions of China. Although a variety of chemical constituents, including alkaloids, flavonoids, and megastigmoids, have been isolated from *H. japonica*, the investigation of saponins in *H. japonica* has not been reported until now. *Methods*: Various separation techniques, including polyporous resin column chromatography, silica gel column chromatography and hemi-preparative HPLC were applied to the isolation of triterpenoid saponins, and chemical methods such as acid hydrolysis and spectroscopic methods including HRESIMS and NMR were applied to their structure elucidation, and the XTT reduction method was used to assay cytotoxicity. *Results*: Two new triterpenoid saponins, named hylomeconoside A (**1**) and B (**2**) which were identified as 3-*O*-β-d-galactopyranosyl-(1→2)-β-d-glucuronopyranosyl-gypsogenin-28-*O*-β-d-xylopyranosyl-(1→3)-β-d-xylopyranosyl-(1→4)-α-l-rhamnopyranosyl-(1→2)-β-d-quinovopyranoside (**1**) and 3-*O*-β-d-galactopyranosyl-(1→2)-β-d-glucuronopyranosyl-gypsogenin-28-*O*-β-d-xylopyranosyl-(1→3)-β-d-xylopyranosyl-(1→4)-α-l-rhamnopyranosyl-(1→2)-α-l-arabinopyranoside (**2**), and two known triterpenoid saponins identified as dubioside C (**3**) and lucyoside P (**4**) on the basis of spectroscopic and chemical evidence, were isolated from *H. japonica.* Compound **1** exhibited moderate cytotoxicity on MGC-803 and HL-60 cells, with IC_50_ values of 43.8 and 32.4 μg·mL^−1^, respectively. *Conclusions:* Compounds **1** and **2** are new saponins, and **1** is considered to be one of the antitumor principles in this plant. This is the first time that triterpenoid saponins have been isolated from plants of the *Papaveraceae* family.

## 1. Introduction

**Figure 1:**
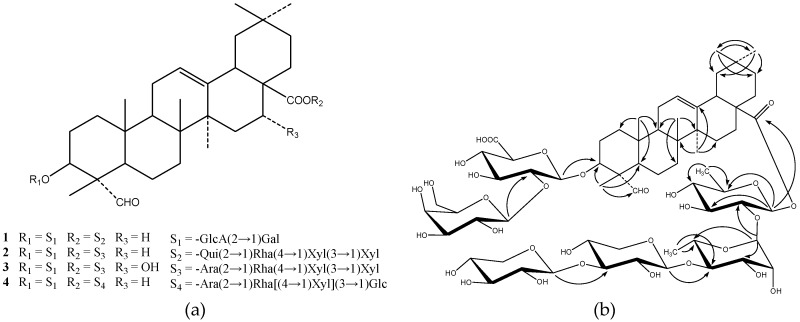
(**a**) Chemical structures of compounds **1**–**4**; (**b**) Key HMBC correlations of compound **1** (arrows point from proton to carbon).

*Hylomecon japonica* (Thunb.) Prantl et Kundig (*Papaveraceae*) is a perennial plant widely distributed in the northeast, central and east regions of China. Its roots have been used as a traditional Chinese medicine for the treatment of rheumatism and injuries [1]. Although a variety of chemical constituents, including alkaloids, flavonoids, and megastigmoids, were previously isolated from *H. japonica*, and pharmacological studies on its anti-inflammatory properties, antibacterial activities, and antitumor action have been reported [2,3,4,5,6,7], the investigation of saponins in *H. japonica* has not been reported until now. As part of our study on the plants of *Papaveraceae* family, we have been carrying out an investigation of saponins in *H. japonica*. In this paper, we now report the isolation and characterization of two new triterpenoid saponins, named hylomeconoside A (**1**) and B (**2**), which were identified as 3-*O*-β-d-galactopyranosyl-(1→2)-β-d-glucuronopyranosyl-gypsogenin-28-*O*-β-d-xylo-pyranosyl-(1→3)-β-d-xylopyranosyl-(1→4)-α-l-rhamnopyranosyl-(1→2)-β-d-quinovopyranoside (**1**) and 3-*O*-β-d-galactopyranosyl-(1→2)-β-d-glucuronopyranosyl-gypsogenin-28-*O*-β-d-xylopyranosyl-(1→3)-β-d-xylopyranosyl-(1→4)-α-l-rhamnopyranosyl-(1→2)-α-l-arabinopyranoside (**2**), and two known triterpenoid saponins identified as dubioside C (**3**) [8], and lucyoside P (**4**) [9] (Figure 1) on the basis of spectroscopic and chemical evidence.

## 2. Results and Discussion

The crude saponins prepared from the 70% EtOH extract of *H. japonica* herbs through a D101 polyporous resin column were subjected to silica gel chromatographies and semi-preparative HPLC to yield two new triterpenoid saponins, named as hylomeconoside A (**1**) and B (**2**), and two known triterpenoid saponins identified as dubioside C (**3**) [8] and lucyoside P (**4**) [9] on the basis of spectroscopic and chemical evidence.

Hylomeconoside A (**1**) was obtained as a white amorphous solid. The molecular formula of **1** was determined as C_64_H_100_O_31_ by high-resolution HRESI-MS which indicated a [M + H]^+^ ion at *m*/*z* 1365.6318. Acid hydrolysis of **1** afforded an aglycone which was identified as gypsogenin on the basis of the ^1^H- and ^13^C-NMR data [10]. The sugars obtained from the saponin hydrolysate were identified as d-xylose, l-rhamnose, d-quinovose, d-glucuronic acid and d-galactose based on GC analysis of their chiral derivatives. The ^1^H-NMR spectrum revealed signals due to six quaternary methyls at δ_H_ 0.68, 0.76, 0.79, 0.90, 1.14, 1.28, an olefinic proton at δ_H_ 5.30 (br. s), an aldehyde proton at δ_H_ 9.83 (s), and six anomeric protons at δ_H_ 6.34 (br s), 5.98 (d, *J* = 8.0 Hz), 5.11 (d, *J* = 7.5 Hz) , 5.08 (d, *J* = 7.4 Hz), 4.94 (d, *J* = 6.8 Hz), and 4.72 (d, *J* = 6.3 Hz). The ^13^C-NMR spectrum displayed signals due to six quaternary carbon at δ_C_ 30.9, 36.4, 40.4, 42.4, 47.3, and 55.2, an oxygen-bearing methine carbon at δ_C_ 83.5, a set of olefinic carbons at δ_C_ 122.7 and 144.2, an ester carbonyl carbon at δ_C_ 176.7, an aldehyde carbon at δ_C_ 210.2, and six anomeric carbons at δ_C_ 94.5, 101.4, 103.4, 105.9, 106.2, and 106.9. All the above spectral information suggested that the aglycone moiety of **1** was gypsogenin and the chemical shift of the carbonyl C-28 (δ_C_ 176.7) and the deshielded carbon C-3 (δ_C_ 83.5) of gypsogenin indicated that **1** was the 3,28-bisdesmoside of gypsogenin, having six monosaccharide units.

The six anomeric proton signals at δ_H_ 6.34 (br s), 5.98 (d, *J* = 8.0 Hz), 5.11 (d, *J* = 7.5 Hz), 5.08 (d, *J* = 7.4 Hz), 4.94 (d, *J* = 6.8 Hz), and 4.72 (d, *J* = 6.3 Hz) were correlated with anomeric carbon signals at δ_C_ 101.4, 94.5, 106.2,105.9, 106.9, 103.4, respectively, and the analysis of ^1^H-^1^H COSY, HMQC, HMBC, DEPT NMR and spin-spin couplings in ^1^H-NMR of **1** allowed the identification of one α-l-rhamnopyranosyl (Rha), one β-d-quinovopyranosyl (Qui), one β-d-glucuronopyranosyl (GlcA), one β-d-galactopyranosyl (Gal) and two β-d-xylopyranosyl (Xyl) moieties. The β-anomeric configurations for d-xylopyranosyl, d-quinovopyranosyl, d-glucuronopyranosyl and d-galactopyranosyl moieties were determined by their large ^3^J_H1-H2_ coupling constants of 6–8 Hz and the α-anomeric configuration for l-rhamnopyranosyl unit was determined by its small ^3^J_H1-H2_ coupling constant. 

One Xyl unit, Xyl^a^, identified starting from anomeric signals at δ_H_ 5.08 (H-1 of Xyl^a^) and δ_C_ 105.9 (C-1 of Xyl^a^), was identified to be in the terminal position, as observed by its ^13^C-NMR chemical shifts. Another Xyl unit, Xyl^b^, identified starting from anomeric signals at δ_H_ 4.94 (H-1 of Xyl^b^) and δ_C_ 106.9 (C-1 of Xyl^b^), was substituted at the position of C-3 of Xyl^b^ based on the deshielding of C-3 of Xyl^b^ (δ_C_ 87.2), and the Xyl^a^ unit was attached to this position based on the long-range correlation observed in the HMBC experiment between signals at δ_H_ 5.08 (H-1 of Xyl^a^) and δ_C_ 87.2 (C-3 of Xyl^b^). The methyl doublet at δ_H_ 1.66 (3H, d, *J* = 6.4 Hz) and the typical broad single of anomeric proton at δ_H_ 6.34 (br s) were characteristic of Rha unit, and the deshielding of C-4 of Rha (δ_C_ 85.3) indicated a substitution at the position of C-4 of Rha, and the long-range correlation between signals at δ_H_ 4.94 (H-1 of Xyl^b^) and δ_C_ 85.3 (C-4 of Rha) indicated that Xyl^b^ was attached to the position of C-4 of Rha. The Qui unit was identified starting from the deshielded anomeric proton at δ_H_ 5.98 (*J* = 8.0 Hz) and characterized by its methyl doublet at δ_H_ 1.42 (3H, d, *J* = 6.0 Hz). The deshielding of anomeric proton and the chemical shift of anomeric carbon at δ_C_ 94.5 suggested that Qui was attached by an ester linkage to the C-28 carboxylic group of the aglycone, which was further confirmed by the long-range correlation observed in the HMBC experiment between signals at δ_H_ 5.98 (H-1 of Qui) and δ_C_ 176.7 (C-28 of gypsogenin). Qui was substituted at the position of C-2 of Qui as observed by its deshielded H-2 of of Qui (δ_H_ 4.23) and the long-range correlation observed in the HMBC experiment between signals of the Rha anomeric proton (δ_H_ 6.34) and C-2 of Qui (δ_C_ 76.6). Therefore, the sequencing of the ester chain was obtained by analysis of HMBC experiment which showed cross-peaks between H-1 of Rha (δ_H_ 6.34) and C-2 of Qui (δ_C_ 76.6), between H-1 of Xyl^b^ (inner xylose) (δ_H_ 4.94) and C-4 of Rha (δ_C_ 85.3), and between H-1 of Xyl^a^ (the terminal xylose) (δ_H_ 5.08) and C-3 of Xyl^b^ (δ_C_ 87.2), and thus the ester chain was a tetrasaccharide, β-d-xylopyranosyl-(1→3)-β-d-xylopyranosyl-(1→4)-α-l-rhamnopyranosyl-(1→2)-β-d-quinovopyranosyl unit.

The Gal unit, identified starting from anomeric signals at δ_H_ 5.11 (H-1 of Gal) and δ_C_ 106.2 (C-1 of Gal), was identified to be in terminal position, as observed by its ^13^C-NMR chemical shifts. Starting from the anomeric proton at δ_H_ 4.72 (d, *J* = 6.3 Hz), a GlcA unit was identified with its carbonyl C-6 at δ_C_ 173.7. The deshielding of C-2 of GlcA (δ_C_ 82.1) indicated a substitution of glucuronic acid. Observation of long-range proton-carbon correlations in the HMBC spectrum between the anomeric proton of GlcA (δ_H_ 4.49) and C-3 of gypsogenin (δ_C_ 83.5) and between the anomeric proton of Gal (δ_H_ 5.11) and C-2 of GlcA (δ_C_ 82.1) indicated a disaccharide chain attached at C-3 of gypsogenin, β-d-galactopyranosyl-(1→2)-β-d-glucuronopyranosyl unit.

The complete assignment of the signals of **1** was based on DEPT ^13^C-NMR and 2D NMR of ^1^H-^1^H COSY, HMQC and HMBC. All the data of ^1^H-, ^13^C-NMR and 2D-NMR of **1** see Table 1, and the key correlations in HMBC NMR and the structure of **1** see Figure 1. In conclusion, compound **1** was identified as 3-*O*-β-d-galactopyranosyl-(1→2)-β-d-glucuronopyranosyl-gypsogenin-28-*O*-β-d-xylo-pyranosyl-(1→3)-β-d-xylopyranosyl-(1→4)-α-l-rhamnopyranosyl-(1→2)-β-d-quinovopyranoside.

Hylomeconoside B (**2**) was obtained as a white amorphous solid. The molecular formula of **2** was determined as C_63_H_98_O_31_ by high-resolution HRESI-MS which showed a [M + H]^+^ ion at *m*/*z* 1351.6151. Acid hydrolysis of **2** afforded an aglycone which was identified as gypsogenin on the basis of the ^1^H- and ^13^C-NMR spectra [10]. The sugars obtained from the saponin hydrolysate were identified as l-arabinose, d-xylose, l-rhamnose, d-glucuronic acid and d-galactose based on GC analysis of their chiral derivatives. In comparative analysis of the ^13^C-NMR data of **2** with those of **1** and **3**, it was found that the spectra data of the aglycone moiety of **2** are same as those of **1**, and the spectra data of the sugar moiety of **2** are consistent with those of **3** (Table 2), which suggested that the aglycone of **2** is gypsogenin and the structure of the sugar moiety is same as those of **3**. By further analysis of spectral data with the similar method as above, **2** was identified as 3-*O*-β-d-galactopyranosyl-(1→2)-β-d-glucuronopyranosyl-gypsogenin-28-*O*-β-d-xylopyranosyl-(1→3)-β-d-xylopyranosyl-(1→4)-α-l-rhamnopyranosyl-(1→2)-α-l-arabinopyranoside.

Dubioside C (**3**), one of active constituents of *Thladianthae dubiae* roots, has been reported to possess analgesic and anti-inflammatory effects [11,12,13] and lucyoside P (**4**), one of active constituntes of *Luffa cylindrica* fructus, has beneficial effects on intelligence [14], therefore we have only carried out an activity assay on the new saponins **1** and **2**. The cytotoxic activities of compounds **1** and **2** on MGC-803 (human gastric cancer), HL-60 (human promyelocytic leukemia), BEL-7404 (humanhepatoma carcinoma), MCF-7 (human breast cancer), and SPC-A1 (lung adenocarcinoma) cells were assessed by the XTT (2,3-Bis-(2-methoxy-4-nitro-5-sulfophenyl)-2*H*-tetrazolium-5-carboxanilide) reduction method.

The results (Table 3) showed that compound **1** exhibited moderate cytotoxicity on MGC-803 and HL-60, with IC_50_ values of 43.8 and 32.4 μg·mL^−1^, respectively. Therefore, compound **1** is considered to be one of the antitumor principles in this plant.

## 3. Materials and Methods 

### 3.1. General Information

NMR spectra were recorded on an AV-400 spectrometer (Bruker Corporation, Faellanden, Switzerland). HRESI-MS were recorded on a Bruker microOTOF-Q II mass spectrometer (Bruker Corporation, Bremen, Germany). Optical rotations were measured with a HORIBA SEPA-300 high-sensitive polarimeter (Horiba Ltd, Kyoto, Japan). HPLC was performed on a Shimadzu LC-10A system equipped with a SPD-10A detector (Shimadzu Corporation, Kyoto, Japan) and a Gemini 5 μm C18 110A column (250 mm × 10.00 mm, 5 μm, Phenomenex, Torrance, CA, USA). GC was performed an Agilent 7820A gas chromatograph with a quartz capillary column (30 mm × 0.32 mm × 0.25 μm, Agilent Technologies Inc., Santa Clara, CA, USA); detection, FID. Column chromatography was performed on silica gel (200–300 mesh, Qingdao Marine Chemical Inc., Qingdao, China), D101 polyporous resin (Tianjin Pesticide Co., LTD., Resin Branch, Tianjin, China). TLC was performed on glass precoated silica gel GF_254_ plates (Qingdao Haiyang Chemical Co., Ltd, Qingdao, China), detection under UV light or by heating after spraying with 10% H_2_SO_4_ in 95% EtOH. Distilled water was purchased from Hangzhou Wahaha Group Co., Ltd. (Hongzhou, China). Acetonitrile of chromatographic grade for HPLC was purchased from Fisher Scientific (Fair Lawn, NJ, USA). Other chemicals and reagents of analytical grade were from Beijing Chemical Works (Beijing, China).

The bioactivities were measured on a DNM-9602 enzyme immunoassay spectrophotometer (Beijing, China). RPMI 1640 medium was purchased from HyClone (Logan, UT, USA), PBS from Gibco company (Carlsbad, CA, USA), fetal bovine serum (FBS) from Zhejiang Tianhang Biotechnology Co., Ltd. (Hangzhou, China), XTT (2,3-Bis-(2-methoxy-4-nitro-5-sulfophenyl)-2H- tetrazolium-5-carboxanilide) and phenazine methosulphate from Sigma-Aldrich Shanghai Trading Co. Ltd. (Shanghai, China).

The *Hylomecon japonica* herb was collected in the Changchun District of Jilin Province, China. They were identified by Jing-min Zhang of the School of Pharmaceutical Sciences, Jilin University. A voucher specimen (No. 2015062001) is deposited at the Herbarium of the School of Pharmaceutical Sciences, Jilin University.

### 3.2. Extraction and Isolation

Two kg of air-dried whole *Hylomecon japonica* herbs were extracted twice with 20 L of 70% aqueous ethanol solution (*v*/*v*) at room temperature. The extraction solution was concentrated under reduced pressure to remove ethanol, and the water concentrate was filtered and then passed through a D101 polyporous resin column eluting successively with H_2_O, 30% EtOH, 50% EtOH, 70% EtOH, and 95% EtOH. The crude saponin extracts were obtained from 50% aqueous ethanol eluate by vacuum distillation recovery and used for the next experiments. The crude saponin extracts were chromatographed on silica gel columns repeatedly eluted with EtOAc–MeOH–H_2_O (7:3:0.5) and futher purified by semi-preparative HPLC using acetonitrile and 0.1% formic acid solution in water as the mobile phase and the eluate was monitored at 207 nm, to yield compounds **1** (60 mg), **2** (50 mg), **3** (30 mg), and **4** (30 mg).

### 3.3. Characterization

Compound **1**: White amorphous solid, ${\left[\alpha \right]}_{D}^{25}$ +5.0°(c 0.5, pyridine). HRESIMS, *m*/*z*: 1365.6318 [M + H]^+^; calcd for C_64_H_101_O_31_, 1365.6327. ^1^H-NMR (400 MHz, C_5_D_5_N) and ^13^C-NMR (100 MHz, C_5_D_5_N): see Table 1.

Compound **2**: White amorphous solid, ${\left[\alpha \right]}_{D}^{25}$ −15.0°(c 0.5, pyridine). HRESIMS, *m*/*z*: 1351.6151 [M + H]^+^; calcd for C_63_H_99_O_31_, 1351.6165. ^1^H-NMR (400 MHz, C_5_D_5_N) δ: 0.67 (3H, s, H-25), 0.80 (3H, s, H-26), 0.87 (6H, s, H-30, H-29), 1.12 (3H, s, H-27), 1.29 (3H, s, H-24), 1.61 (3H, d, *J* = 4.8, Rha-6), 3.13 (1H, m, H-18), 3.34 (1H, t-like, *J* = 10.0 Hz, Xyl(inner)-5α), 3.55 (1H, t-like, *J* = 10.0 Hz, Xyl(terminal)-5α), 4.71 (1H, d, *J* = 6.8 Hz, GluA-1), 5.01 (1H, d, *J* = 6.8 Hz, Xyl(inner)-1), 5.08 (1H, d, *J* = 7.2 Hz, Gal-1), 5.10 (1H, d, *J* = 7.6 Hz, Xyl(terminal)-1), 5.29 (1H, br. s, H-12), 5.66 (1H, br. s, Rha-1), 6.34 (1H, br. s, Ara-1), 9.82 (1H, s, H-23). ^13^C-NMR (100 MHz, C_5_D_5_N): see Table 2.

Compound **3**: White amorphous solid, ${\left[\alpha \right]}_{D}^{25}$ −29.2°(c 0.5, pyridine). HRESIMS, *m*/*z*: 1367.6101 [M + H]^+^; calcd for C_63_H_99_O_32_, 1367.6114. ^1^H-NMR (400 MHz, C_5_D_5_N) δ: 0.70 (3H, s, H-25), 0.90 (6H, s, H-26, H-29), 1.02 (3H, s, H-30), 1.28 (3H, s, H-27), 1.57 (3H, s, H-24), 1.65 (3H, d, *J* = 4.8, Rha-6), 3.44 (1H, m, H-18), 3.31 (1H, t-like, *J* = 10.0 Hz, Xyl(inner)-5α), 3.54 (1H, t-like, *J* = 10.0 Hz, Xyl(terminal)-5α), 4.68 (1H, d, *J* = 6.8 Hz, GluA-1), 4.98 (1H, d, *J* = 6.8 Hz, Xyl(inner)-1), 5.05 (1H, d, *J* = 7.2 Hz, Gal-1), 5.10 (1H, d, *J* = 7.6 Hz, Xyl(terminal)-1), 5.47 (1H, br. s, H-12), 5.62 (1H, br. s, Rha-1), 6.35 (1H, br. s, Ara-1), 9.76 (1H, s, H-23). ^13^C-NMR (100 MHz, C_5_D_5_N): see Table 2.

Compound **4**: White amorphous solid, ${\left[\alpha \right]}_{D}^{25}$ −10.0°(c 0.5, pyridine). HRESIMS, *m*/*z*: 1381.6258 [M + H]^+^; calcd for C_64_H_101_O_32_, 1381.6270. ^1^H-NMR (400 MHz, D_2_O-C_5_D_5_N) δ: 0.90 (3H, s, H-25), 0.98 (3H, s, H-26), 1.03 (3H, s, H-29), 1.04 (3H, s, H-30), 1.26 (3H, s, H-27), 1.45 (3H, s, H-24), 1.67 (3H, d, *J* = 4.8, Rha-6), 3.17 (1H, m, H-18), 3.57 (1H, t-like, *J* = 10.0 Hz, Xyl(terminal)-5α), 4.82 (1H, d, *J* = 7.2 Hz, GluA-1), 5.09 (1H, d, *J* = 8.0 Hz, Glu-1), 5.18 (1H, d, *J* = 7.6 Hz, Gal-1), 5.29 (1H, d, *J* = 8.0 Hz, Xyl-1), 5.45 (1H, br. s, H-12), 5.57 (1H, br. s, Rha-1), 6.23 z, Ara-1), 9.78 (1H, s, H-23). ^13^C-NMR (100 MHz, D_2_O-C_5_D_5_N): See Table 2.

### 3.4. Acid Hydrolysis and Sterochemistry of Sugars of ***1*** and ***2***

Compounds **1** and **2** (each 20 mg) were hydrolyzed with 2 M HCl (5 mL) at 90 °C for 4 h. The reaction mixture was extracted with EtOAc (3 × 5 mL), washed with H_2_O, and evaporated to dryness. The EtOAc extract was purified by silica gel column using a gradient of CHCl_3_-MeOH (1:0–95:5) to the aglycones of **1** and **2** which were determined to be gypsogenin by comparison of their spectral data with those of an authentic sample. Aglycone: White amorphous solid; ^13^C-NMR (100 MHz, C_5_D_5_N) δ: 207.7 (C-23), 180.0 (C-28), 144.8 (C-13), 122.2 (C-12), 71.6 (C-3), 56.2(C-4), 48.0 (C-5), 47.9 (C-9), 46.6 (C-17), 46.5 (C-19), 42.2 (C-14), 41.9 (C-18), 40.0 (C-8), 38.4 (C-1), 36.2 (C-10), 34.2 (C-21), 33.2 (C-29), 32.8 (C-7), 32.5 (C-22), 30.9 (C-20), 28.3 (C-15), 26.1 (C-27), 27.0 (C-2), 23.8 (C-16, C-30), 23.7 (C-11), 21.0 (C-6), 17.3 (C-26), 15.7 (C-25), 9.6 (C-24); ^1^H-NMR (400 MHz, C_5_D_5_N) δ: 0.94 (3H, s), 0.97 (6H, s), 1.12 (6H, s), 1.23 (6H, s), 1.41 (6H, s), 3.02 (1H, m), 3.90(1H, m), 5.29 (1H, brs), 9.66 (1H, m). The H_2_O layer was concentrated under reduced pressure to dryness, to give a residue of the sugar fraction. The residue was dissolved in pyridine (2 mL), l-cysteine methyl ester hydrochloride (1.5 mg) was added, and the mixture was heated at 60 °C for 1 h. Trimethylsilylimidazole (1.5 mL) was added, and the mixture was heated at 60 °C for another 0.5 h. An aliquot (4 μL) of the supernatant was subjected to GC analysis under the following conditions: column temp 180–280 °C at 3 deg/min, carrier gas N_2_ (1 mL/min), injector and detector temp 250 °C, split ratio 1:50. The configurations of monosugars for **1** and **2** were determined by comparison of the retentions times (t_R_) of the corresponding derivatives with standard l-arabinose (t_R_ 10.748 min), d-xylose (t_R_ 11.496 min), l-rhamnose (t_R_ 12.162 min), d-quinovose (t_R_ 13.648 min), d-glucuronic acid (t_R_ 15.814 min) and d-galactose (t_R_ 18.026 min). **1** yielded d-xylose, l-rhamnose, d-quinovose, d-glucuronic acid and d-galactose, and **2** gave l-arabinose, d-xylose, l-rhamnose, d-glucuronic acid and d-galactose.

### 3.5. Cytotoxicity Assay

The procedure for the cytotoxic assay was performed according to the XTT (2,3-bis-(2-methoxy-4-nitro-5-sulfophenyl)-2*H*-tetrazolium-5-carboxanilide) reduction method. In this study, MGC-803 (human gastric cancer), HL-60 (human promyelocytic leukemia), BEL-7402 (humanhepatoma carcinoma), MCF-7 (human breast cancer), and SPC-A1 (lung adenocarcinoma ) cell lines were used. Compounds **1** and **2** was dissolved at a concentration of 10 mg·mL^−1^ in phosphate-buffered saline (PBS) (as a stock solution), filtered through a filter 0.22 μm (Millipore, Bedford, MA, USA) and stored at 4 °C. The stock solution was diluted to 160, 80, 40, 20, 10 μg·mL^−1^ before the cytotoxic assay. 

Logarithmic-phase growing cells were diluted into 5 × 10^4^ cells·mL^−1^, seeded in in 96-well plates with 100 μL per well, and incubated in RPMI 1640 medium supplemented with 10% fetal bovine serum, 100 U·mL^−1^ penicillin, 100 μg·mL^−1^ streptomycin at 37 °C in a humidified atmosphere with 5% CO_2_. Blank control wells were added 100 μL of cultrure medium, and maintained under the same conditions. After 24 h, 100 μL of the above culture medium containing different concentrations of **1** and **2** and normal culture medium (untreated control group) were added, and incubated for 72 h. For the analysis of cytotoxicity, 50 μL (1 mg·mL^−1^) XTT containing 0.15 mg·mL^−1^ phenazine methosulphate was added to each well, and cells were incubated for 3 h at 37 °C. The absorbances (A) of the produced formazan were measured by a DNM-9602 enzyme immunoassay spectrophotometer at 450 nm. Three replicate well were used for each analysis. The cell inhibitory rate (%) = (A_sample_ − A_blank_)/(A_untreated_ − A_blank_) × 100. The concentration of compound producing 50% of cell inhibitory rate (IC_50_) was calculated by using SPSS version 20.0 (International Business Machines Corporation, Armonk, NY, USA).

## 4. Conclusions

Two new triterpenoid saponins, named hylomeconoside A (**1**) and B (**2**), along with two known triterpenoid saponins identified as dubioside C (**3**), and lucyoside P (**4**), have been isolated from an ethanolic extract of *Hylomecon japonica* herbs. It is well known that alkaloids are main constituents of the plants of *Papaveraceae* family and until now besides alkaloids, flavonoids, megastigmane derivatives, volatile oil, furan derivatives, triterpenes and sterols have also reported, but saponins have not been reported [15,16,17]. This is the first time that saponins have been isolated from plants of the *Papaveraceae* family. Compound **1** with the IC_50_ values of 43.8 μg·mL^−1^ on MGC-803 and 32.4 μg·mL^−1^ on HL-60 is considered to be one of the antitumor principles in this plant.

## Figures and Tables

**Table 1:** The spectral data of ^1^H-NMR (400 MHz), ^13^C-NMR (100 MHz), and 2D-NMR of ^1^H-^1^H COSY and HMQC of compound **1** in C_5_D_5_N.

Aglycone Moiety				Sugar Moiety			
Position	δ_H_ (*J* in Hz)	^1^H-^1^H COSY	δ_C_ HMQC	Position	δ_H_ (*J* in Hz)	^1^H-^1^H COSY	δ_C_ HMQC
1α	0.69 m	H-1β, H-2β	38.3	3-O-			
1β	1.26 m	H-1α		GlcA-1	4.72 d (6.3)	H-2	103.4
2α	2.04 m	H-2β, H-3	25.2	2	4.06 m	H-1, H-3	82.1
2β	1.73 m	H-2α, H-1α, H-3		3	4.12 m	H-2, H-4	77.0
3	3.90 m	H-2α, H-2β	83.5	4	4.28 m		73.2
4			55.2	5	4.27 m		77.9
5	1.25 m	H-6β	48.7	6			173.7
6α	1.26 m	H-6β	20.8	Gal-1	5.11 d (7.5)	H-2	106.2
6β	1.01 m	H-6α, H-7α		2	4.42 m	H-1, H-3	74.4
7α	1.91 m	H-6β, H-7β	32.8	3	3.99 m	H-2	75.0
7β	1.65 m	H-7α		4	4.45 m		70.3
8			40.4	5	3.98 m	H-6	77.0
9	1.52 m	H-11α, H-11β	48.0	6	4.40 m	H-5	62.2
10			36.4	28-O-			
11α	1.71 m	H-9, H-11β	23.8	Qui-1	5.98 d (8.0)	H-2	94.5
11β	1.79 m	H-11α, H-9		2	4.23 t (8.0)	H-1, H-3	76.6
12β	5.30 br.s	H-11α, H-11β	122.7	3	4.05 t (8.4)	H-2, H-4	79.3
13			144.2	4	3.55 t (8.8)	H-3, H-5	76.9
14			42.4	5	3.71 m	H-4, H-6	73.9
15α	1.33 m	H-15β	28.6	6	1.42 d (6.0)	H-5	18.5
15β	1.90 m	H-15α		Rha-1	6.34 br. s	H-2	101.4
16α	2.04 m	H-16β	23.9	2	4.69 br. s	H-1, H-3	71.8
16β	1.76 m	H-16α		3	4.54 dd (9.2, 2.8)	H-2, H-4	72.7
17			47.3	4	4.23 dd (8.4, 9.2)	H-3, H-5	85.3
18	3.02 m	H-19α, H-19β	42.3	5	4.36 m	H-4, H-6	68.3
19α	1.63 m	H-18, H-19β	46.4	6	1.66 d (6.4)	H-5	18.7
19β	1.09m	H-19α, H-18		Xyl^b^-1	4.94 d (6.8)	H-2	106.9
20			30.9	2	3.89 m	H-1, H-3	75.2
21α	1.20 m		34.1	3	3.91 m	H-2, H-4	87.2
21β	1.03 m			4	3.97 m	H-3, H-5α	69.0
22α	1.44 m		32.5	5α	3.37 t (10.4)	H-4, H-5β	67.1
22β	1.42 m			5β	4.10 m	H-4, H-5α	
23	9.83 s		210.2	Xyl^a^-1	5.08 (d, 7.4)	H-2	105.9
24	1.28 s		11.1	2	3.93 (m)	H-1	75.5
25	0.68 s		15.9	3	4.02 (m)		78.2
26	0.90 s		17.6	4	4.03 (m)	H-5α	71.0
27	1.14 s		26.1	5α	3.54 t (9.6)	H-4, H-5β	67.4
28			176.7	5β	4.15 m	H-4, H-5α	
29	0.76 s		33.3				
30	0.79 s		23.9				

**Table 2:** The ^13^C-NMR data of compound **2**–**4** in C_5_D_5_N.

C No.	2	3	4	C No.	2	3	4
1	38.2	38.3	38.3	C-3-O-sugars			
2	25.0	24.9	25.1	GlcA-1	103.3	103.0	103.1
3	83.5	83.3	84.7	2	82.4	82.3	80.0
4	55.2	55.2	55.9	3	77.2	77.2	76.6
5	48.4	48.5	48.3	4	73.3	73.3	72.4
6	20.6	20.6	20.8	5	77.9	77.9	77.3
7	32.8	32.9	32.8	6	173.7	173.7	176.4
8	40.2	40.4	40.3	Gal-1	106.4	106.3	104.4
9	47.9	47.1	48.0	2	74.6	74.6	73.1
10	36.3	36.4	36.3	3	75.0	75.0	74.3
11	23.3	23.9	23.4	4	69.9	70.3	69.8
12	122.8	122.6	123.0	5	77.2	77.2	77.3
13	144.3	144.6	144.4	6	62.2	62.3	62.2
14	42.2	42.2	42.4	C-28-O-sugars			
15	28.4	36.2	28.4	Ara-1	93.6	93.6	93.6
16	23.9	74.1	23.9	2	75.1	75.2	75.9
17	47.4	49.6	47.7	3	69.9	69.9	69.9
18	41.8	41.4	41.9	4	66.2	66.1	66.4
19	46.3	47.1	46.5	5	63.1	63.1	63.1
20	31.0	31.1	31.0	Rha-1	101.0	101.1	101.2
21	34.2	36.1	34.2	2	71.9	72.0	71.2
22	32.6	32.3	32.6	3	72.8	72.8	82.2
23	209.9	209.6	211.8	4	84.0	83.5	78.6
24	11.1	11.0	10.8	5	68.6	68.6	69.0
25	15.8	15.9	15.9	6	18.5	18.5	18.7
26	17.5	17.6	17.6	Xyl(inner)-1	106.6	106.3	
27	26.2	27.3	26.3	2	75.2	75.1	
28	176.4	176.0	177.6	3	87.1	87.2	
29	33.3	33.4	33.4	4	69.1	69.1	
30	23.8	24.9	23.9	5	67.0	67.0	
				Xyl(terminal)-1	106.4	106.1	104.6
				2	75.4	75.4	75.2
				3	78.3	78.3	76.6
				4	71.0	71.0	70.8
				5	67.4	67.4	66.5
				Glc(terminal)-1			105.4
				Glc-2			72.9
				Glc-3			77.5
				Glc-4			70.2
				Glc-5			77.8
				Glc-6			62.0

**Table 3:** The cytotoxicity of compouds **1** and **2** assayed by XTT assay (n = 3).

Compds.	Concentration (μg·mL^−l^)	Inhibitory Rate, Mean ± SD (%)				
		MGC-803	HL-60	BEL-7402	MCF-7	SPC-A1
**1**	10	9.3 ± 1.3 ^1^	11.3 ± 1.6	0	0	0
	20	18.5 ± 1.1	19.7 ± 1.5	0	0	0
	40	43.2 ± 1.6	58.4 ± 1.3	0	0	0
	80	75.0 ± 2.3	91.7 ± 2.1	0	0	25.1 ± 1.2
	160	92.1 ± 1.7	98.2 ± 1.9	22.0 ± 1.7	25.2 ± 1.8	40.1 ± 2.2
**2**	10	0	0	0	0	0
	20	0	0	0	0	0
	40	10.2 ± 1.8	9.2 ± 1.3	0	0	0
	80	18.4 ± 1.6	20.3 ± 1.2	0	0	0
	160	44.3 ± 1.5	55.0 ± 2.2	22.0 ± 1.9	25.20 ± 1.8	23.1 ± 2.1

^1^ Results are expressed as mean ± standard deviation. The IC_50_ values of compound **1** is 43.8 μg·mL^−1^ on MGC-803 and 32.4 μg·mL^−1^ on HL-60.
